# A Novel Cyclodione Coumarin from the Stem Bark of *Mesua beccariana*

**DOI:** 10.3390/molecules16097249

**Published:** 2011-08-25

**Authors:** Gwendoline Cheng Lian Ee, Soek Sin Teh, Siau Hui Mah, Mawardi Rahmani, Yun Hin Taufiq-Yap, Khalijah Awang

**Affiliations:** 1 Department of Chemistry, Faculty of Science, Universiti Putra Malaysia, Serdang, Selangor 43400, Malaysia; E-Mails: judith_teh@hotmail.com (S.S.T.); small_horse_1209@hotmail.com (S.H.M.); mawardi@science.upm.edu.my (M.R.); yap@science.upm.edu.my (Y.H.T.-Y.); 2 Department of Chemistry, Faculty of Science, University of Malaya, Kuala Lumpur 50603, Malaysia; E-Mail: khalijah@um.edu.my

**Keywords:** beccamarin, cyclodione coumarin, *Mesua beccariana*, Clusiaceae

## Abstract

Our ongoing investigations on the stem bark of *Mesua beccariana* afforded a novel cyclodione coumarin, beccamarin, together with two known xanthones, mesuarianone, mesuasinone, two anthraquinones, 4-methoxy-1,3,5-trihydroxy-anthraquinone and 2,5-dihydroxy-1,3,4-trimethoxyanthraquinone and one coumarin, mammea *A/AB*. The structures were elucidated by 1D and 2D NMR and MS techniques.

## 1. Introduction

**Figure 1:**
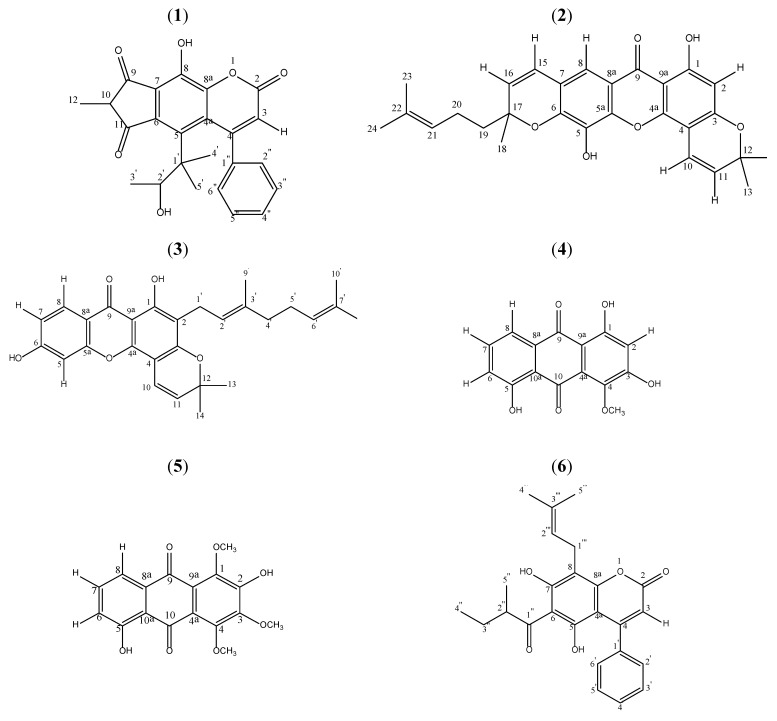
Structures of Compounds. Compounds 2 and 3 were isolated from the hexane extract while compounds 4-6 were found from the ethyl acetate extracts.

*Mesua* is a genus of flowering plants in the family of Clusiaceae, native to tropical southern Asia. Common names for *Mesua* include ironwood and rose chestnut. They are evergreen shrubs or small trees growing up to 13 m tall, with leaves arranged in opposite pairs. The blossoms are white and give off a nice fragrance. Although *Mesua* species have been investigated before, reports on phytochemical constituents of *Mesua* species are few [1]. Phytochemical investigations on the genus show the occurrence of xanthones [2,3,4,5,6], coumarins [2,7,8,9,10], terpenoids [3] and essential oil [8,11]. Our recent study on *Mesua ferrea* identified mesuaferrin A and mesuaferrin B [12]. We report here a novel cyclodione coumarin, beccamarin (**1**). See Figure 1.

## 2. Results and Discussion

Beccamarin (**1**, Figure 1) was isolated as a yellowish solid from the hexane extract of *Mesua beccariana*. Its melting point was 139.0–139.6 °C. The HRESIMS spectrum revealed a molecular ion peak at 405.1361 [M-H]^−^ (calculated 406.1417), which corresponds to a molecular formula of C_24_H_22_O_6_. The UV spectrum supported the coumarin skeleton due to its maxima absorptions at 209 (5.23), 229 (5.27), 281 (5.36) and 348 (5.45). The FTIR spectrum gave absorptions of chelated hydroxyl (3,400 cm^−1^), carbonyl (1,741 cm^−1^), saturated C-H stretch (2971 cm^−1^) and aromatic ring (1,466 and 1605 cm^−1^), which reflected similarity to typical IR bands for coumarins.

The ^1^H-NMR spectrum indicated a monosubstituted phenyl group, deduced by the presence of three sets of triplets at δ 7.38 (*t*, 1H, *J* = 5.7 Hz), 7.37 (*t*, 2H, *J* = 5.7 Hz, overlapped) and 7.30 (*d*, 2H, *J* = 5.7 Hz, overlapped). In the ^13^C-NMR spectrum, the signals at δ 127.2 (C-2” & C-6”, overlapped), 127.7 (C-3” & C-5”, overlapped) and 128.2 (C-4”) belong to the phenyl group. Meanwhile, δ 7.30 (H-2” & H-6”) was correlated to δ 156.8 (C-4) *via* a ^3^*J* correlation and δ 7.37 (H-3” & H-5”) was correlated to δ 139.5 (C-1”) (^3^*J* correlation). All these observations place the phenyl group at C-4 of the main coumarin skeleton.

The ^1^H, ^13^C and HMBC spectra revealed a low field hydroxyl group (δ 14.50, *s*, 1 H). This OH group correlated to δ 164.8 (C-8) *via* a ^2^*J* correlation in the HMBC spectrum. A ^3^*J* correlation to δ 102.6 (C-7) was also observed. Hence, the hydroxyl group was assigned to C-8. The ^1^H, HMQC and HMBC experiments gave one sharp singlet proton signal at δ 5.93 and this has direct connectivity to δ 112.1 (C-3) and long range couplings to δ 102.6 (C-4a), 139.5 (C-1”) and 159.7 (C-2). Thus, this singlet proton was positioned at C-3.

The ^1^H and COSY spectra suggested the occurrence of a methylbutanol moiety with a set of correlated signals which are δ 1.33 (*s*, 3H, H-5’), 1.46 (*d*, 3H, *J* = 6.9, H-3’), 1.58 (*s*, 3H, H-4’) and 4.61 (*q*, 1H, *J* = 6.9, H-2’) with δ 1.46 (*d*, H-3’) coupling to δ 4.61 (*q*, H-2’). The HMQC and HMBC spectrum indicated that δ 4.61 (H-2’) has connectivities with δ 21.0 (C-5’), 25.8 (C-4’), 43.6 (C-1’) and 113.7 (C-5); δ 1.46 (H-3’) correlates with δ 43.6 (C-1’) and 91.9 (C-2’); δ 1.58 (H-4’) has connectivities with δ 21.0 (C-5’), 43.6 (C-1’), 91.9 (C-2’) and 113.7 (C-5); δ 1.33 (H-5’) correlates with δ 25.8 (C-4’), 43.6 (C-1’), 91.9 (C-2’) and 113.7 (C-5). This information justifies a 3-methylbutan-2-ol group to be attached to the coumarin ring at position C-5 (δ 113.7) leaving C-6 and C-7 to form a fused ring. 

**Figure 2:**
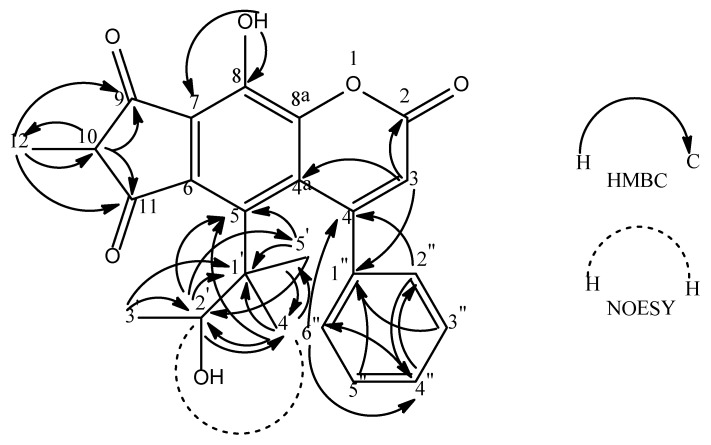
Selected HMBC (^2^*J* and ^3^*J*) and NOESY correlations for compound 1.

In addition, the ^1^H-NMR experiment demonstrated the presence of a multiplet and doublet signal at the upfield region (δ 3.77, 1H and δ 1.15, 3H, respectively). The signal at δ 3.77 (H-10) was directly bonded to δ 39.2 (C-10) whereas the signal at δ 1.15 (H-12) was directly bonded to δ 18.8 (C-12) as seen in the HMQC spectrum. The HMBC spectrum showed H-10 to have connectivities with δ 18.8 (C-12), 209.9 (C-11) and 210.1 (C-9); Meanwhile, H-12 has connectivities with δ 39.2 (C-10), 209.9 (C-11) and δ 210.1 (C-9). This implies the presence of 2-methylcyclopentane-1,3-dione. This substituent group has to be placed at C-6 and C-7 since C-5 carries the 3-methylbutan-2-ol group. The left ring of the coumarin is thus substituted at C-7 and C-6 by the above dione substituent group. (Figure 2). 

Meanwhile, the NOESY experiment shows correlations between H-4’ and H-2’ suggesting that they have a similar configuration. The HMBC and NOESY correlations for **1** are shown in Figure 2. Therefore, compound **1** was identified as 4-phenyl-8-hydroxy-5-(3-methylbutan-2-ol)-(2-methylcyclopentane-1,3-dione)-[4”,5”:6,7]-chromen-2-one.

## 3. Experimental

### 3.1. General

Infrared spectra were measured using the universal attenuated total reflection (UATR) technique on a Perkin-Elmer 100 Series FT-IR spectrometer. EIMS were recorded on a Shimadzu GCMS-QP5050A spectrometer. NMR spectra were obtained on a Unity JEOL 500 MHz FT-NMR spectrometer using CDCl_3_ as solvent and tetramethylsilane (TMS) as internal standard. Ultraviolet spectra were recorded in EtOH on a Shimadzu UV-160A, UV-Visible Recording Spectrophotometer. 

### 3.2. Plant Material

The stem bark of *Mesua beccariana* was collected from the Sri Aman district in Sarawak, Malaysia. The plant material was identified by Associate Professor Dr Rusea Go, Biology Department, Faculty of Science, Universiti Putra Malaysia.

### 3.3. Extraction and Isolation

The three kg of milled, air-dried and powdered sample was defatted with *n*-hexane and extracted successively with dichloromethane, ethyl acetate and methanol. The extracts were dried under reduced pressure using a rotary evaporator to yield hexane (15.6 g), dichloromethane (21.2 g), ethyl acetate (15.8 g) and methanol (80.5 g) extracts. Each of these extracts was chromatographed over a silica gel column using a stepwise gradient system (hexane/chloroform, chloroform/ethyl acetate, and ethyl acetate/methanol). The hexane extract was subjected to vacuum column chromatography over silica gel with a stepwise gradient of hexane/dichloromethane (hexane/CH_2_Cl_2_) and dichloromethane/ethyl acetate (CH_2_Cl_2_/EA). The eluted fraction A (CH_2_Cl_2_/EA-80:20, 3.5 g) was then subjected to a flash column chromatography using hexane/CH_2_Cl_2_ to obtain fraction B (hex/ CH_2_Cl_2_-30:70, 36 mg) to give beccamarin. Beccamarin was crystallized by repeated crystallizations from hexane. The hexane extract provided beccamarin (**1**, 20 mg), mesuarianone (**2**, 110 mg), mesuasinone (**3**, 76 mg) while the ethyl acetate extract gave two anthraquinones, 4-methoxy-1,3,5-trihydroxyanthraquinone (**4**, 9 mg) and 2,5-dihydroxy-1,3,4-trimethoxyanthraquinone (**5**, 8 mg) and a coumarin, mammea *A/AB* (**6**, 11 mg).

### 3.4. Spectral Data

*Beccamarin* (**1**). Yellow solid. UV (EtOH) λ_max_ nm (log ε): 209 (5.23), 229 (5.27), 281 (5.36) and 348 (5.45). IR ν_max_ (cm^-1^): 3400, 2971, 1741, 1605 and 1466. MS *m/z* (rel. int.): 406 [M^+^] (12), 392 (28), 377 (16), 350 (24), 349 (100), 293 (19), 43 (18). For ^1^H and ^13^C-NMR spectra, see Table 1.

*Mesuarianone* (**2**). Yellow solid. UV (EtOH) λ_max_ nm (log ε): 208, 280 and 333.8. IR ν_max_ (cm^−^^1^): 3392, 2971, 2923, 1639, 1574, 1475. MS *m/z* (rel. int.): 460 [M^+^] (39), 445 (100), 377 (89), 361 (10), 347 (11), 323 (12), 203 (8), 181 (61), 91 (6), 77 (5), 69 (27), 55 (9). The ^1^H- and ^13^C-NMR (CDCl_3_) spectral data are consistent with published data [1].

*Mesuasinone* (**3**). Yellow solid. UV (EtOH) λ_max_ nm (log ε): 208, 254, 274 and 334. IR ν_max_ (cm^−^^1^): 3229, 2925, 2865, 1640, 1576, 1496. MS *m/z* (rel. int.): 446 [M^+^] (14), 431 (4), 391 (8), 363 (100), 307 (21), 154 (12), 69 (10). The ^1^H and ^13^C NMR (CDCl_3_) spectral data are consistent with published data[1].

*4-Methoxy-1,3,5-trihydroxyanthraquinone* (**4**). Orange solid. UV (MeOH) λ_max_ nm (log ε): 279, 320, 425, 470 and 485. IR ν_max_ (cm^−^^1^): 3420, 2920, 2860, 1720, 1630, 1470. MS *m/z* (rel. int.): 286 [M^+^] (100), 268 (87), 257 (10), 243 (38), 212 (27), 180 (30). The ^1^H and ^13^C NMR (CDCl_3_) spectral data are consistent with published data[13].

*2,5-Dihydroxy-1,3,4-trimethoxyanthraquinone* (**5**). Orange solid. UV (MeOH) λ_max_ nm (log ε): 218, 276 and 410. IR ν_max_ (cm^−^^1^): 3400, 2920, 2840, 1660, 1630, 1540. MS *m/z* (rel. int.): 330 [M^+^] (100), 315 (60), 312 (5), 297 (20), 287 (22), 272 (24), 227 (20), 58 (23). The ^1^H and ^13^C NMR (CDCl_3_) spectral data are consistent with published data[13]. 

*Mammea A/AB* (**6**). Colourless solid. UV (EtOH) λ_max_ nm (log ε): 283 and 337. IR ν_max_ (cm^−^^1^): 3296, 2930, 1706, 1621. MS *m/z* (rel. int.): 406 [M^+^] (2), 392 (18), 377 (10), 349 (100), 293 (10). The ^1^H and ^13^C NMR (CDCl_3_) spectral data are consistent with published data[10]. 

## 4. Conclusions

A novel cyclodione coumarin, beccamarin (**1**), along with two xanthones, two anthraquinones and another coumarin were isolated from the stem bark of *Mesua beccariana*. 

## Figures and Tables

**Table 1:** ^1^H-NMR (500 MHz, CDCl_3_) and ^13^C-NMR (125 MHz, CDCl_3_) data for beccamarin (**1**).

Position	^1^H(*δ*)	^13^C(*δ*)	HMBC
1	-	-	-
2	-	159.7	-
3	5.93 (*s*, 1H)	112.1	102.6 (C-4a), 139.5 (C-1”), 159.7 (C-2)
4	-	156.8	-
4a	-	102.6	-
5	-	113.7	-
6	-	103.0	-
7	-	102.6	-
8	-	164.8	-
8a	-	156.4	-
9	-	210.1	-
10	3.77 (*m*, 1H)	39.2	18.8 (C-12), 209.9 (C-11), 210.1 (C-9)
11	-	209.9	-
12	1.15 (*d*, 3H)	18.8	39.2 (C-10), 209.9 (C-11), 210.1 (C-9)
1’	-	43.6	-
2’	4.61 (*q*, 1H, 6.9)	91.9	21.0 (C-5’), 25.8 (C-4’), 43.6 (C-1’), 113.7 (C-5)
3’	1.46 (*d*, 3H, 6.9)	14.5	43.6 (C-1’), 91.9 (C-2’)
4’	1.58 (*s*, 3H)	25.8	21.0 (C-5’), 43.6 (C-1’), 91.9 (C-2’), 113.7 (C-5)
5’	1.33 (*s*, 3H)	21.0	25.8 (C-4’), 43.6 (C-1’), 91.9 (C-2’), 113.7 (C-5)
1”	-	139.5	-
2”	7.30 (*d*, 1H, 5.7)	127.2	128.2 (C-4”), 156.8 (C-4)
3”	7.37 (*t*, 1H, 5.7)	127.7	139.5 (C-1”)
4”	7.38 (*t*, 1H, 5.7)	128.2	127.2 (C-2”& C-6”)
5”	7.37 (*t*, 1H, 5.7)	127.7	139.5 (C-1”)
6”	7.30 (*d*, 1H, 5.7)	127.2	128.2 (C-4”), 156.8 (C-4)
8-OH	14.50 (*s*, 1H)	-	102.6 (C-7), 164.8 (C-8)
2’-OH	4.61 (*s*, 1H)	-	-
